# Memory capacity and prioritization in female mice

**DOI:** 10.1038/s41598-023-40976-y

**Published:** 2023-08-28

**Authors:** Qinbo Qiao, Caroline Mairlot, Daniel Bendor

**Affiliations:** Institute of Behavioural Neuroscience (IBN), University College London (UCL), London, WC1H 0AP UK

**Keywords:** Consolidation, Forgetting

## Abstract

Our brain’s capacity for memory storage may be vast but is still finite. Given that we cannot remember the entirety of our experiences, how does our brain select what to remember and what to forget? Much like the triage of a hospital’s emergency room, where urgent cases are prioritized and less critical patients receive delayed or even no care, the brain is believed to go through a similar process of memory triage. Recent salient memories are prioritized for consolidation, which helps create stable, long-term representations in the brain; less salient memories receive a lower priority, and are eventually forgotten if not sufficiently consolidated (Stickgold and Walker in Nat Neurosci 16(2):139–145, 2013). While rodents are a primary model for studying memory consolidation, common behavioral tests typically rely on a limited number of items or contexts, well within the memory capacity of the subject. A memory test allowing us to exceed an animal’s memory capacity is key to investigating how memories are selectively strengthened or forgotten. Here we report a new serial novel object recognition task designed to measure memory capacity and prioritization, which we test and validate using female mice.

## Introduction

What determines whether a memory will be retained long-term or eventually forgotten? When items are presented in a series, memory retention is generally better for the first and last items, a phenomenon commonly referred to as the primacy and recency effect respectively^1–3^. This phenomenon has been widely observed and studied in both humans^3–6^ and other animals, including monkeys^7^, dolphins^8^, pigeons^9,10^ and rats^11–14^, although usually over short delay periods and within a single context or task. Additional factors influencing memory retention are repetition, where additional repetitions or a longer training duration promotes stronger and longer-lasting memories^15^, and salience (or perceived future relevance) which can result from multiple factors including novelty, reward and/or emotion^16–20^.

Here, we describe a serial novel object recognition (sNOR) task in rodents^21^, using a multi-arena behavioural apparatus, providing us with a new tool for investigating how different task factors influence memory retention and forgetting. Importantly, this approach uses novel object recognition, allowing for the quick acquisition of new memories, with learning requiring only a single behavioral session. However, differing from a typically novel object recognition task, this behavioral paradigm uses multiple small behavioral arenas connected in series with entry and exit controlled by the experimenter, with each arena having a different set of novel objects for the animal to learn.

## Results

**Figure 1:**
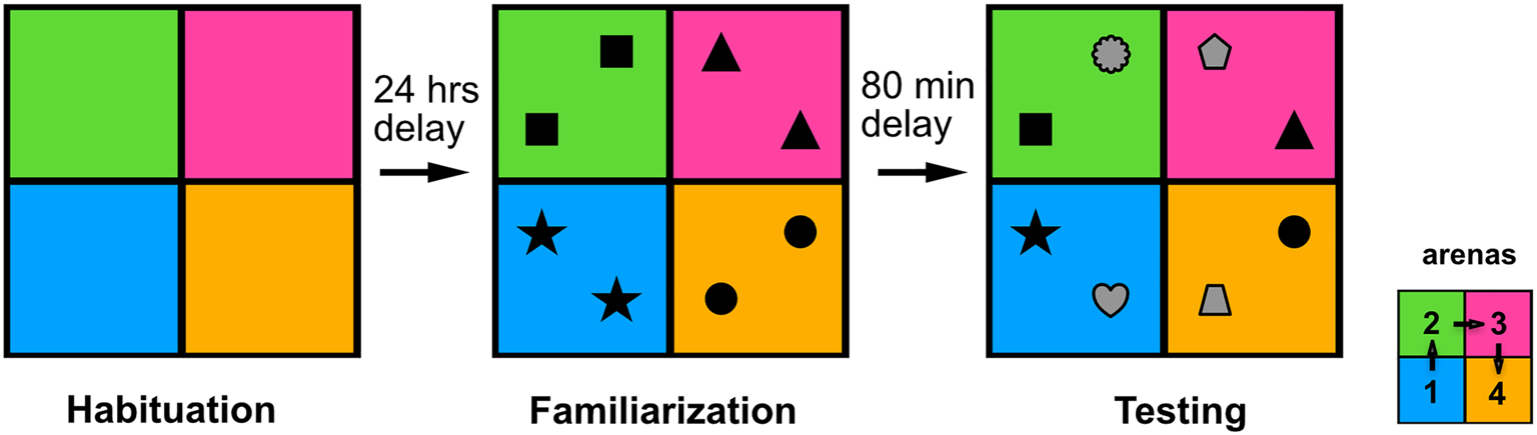
Serial object recognition task design.

We designed a serial object recognition task (Fig. 1), where female mice were trained to remember four distinct novel objects, each associated with a different behavioral arena. The experiment involved three distinct phases (habituation, familiarization, and testing) similar to the standard version of a novel object discrimination task involving a single arena^22^. In the habituation phase, mice received a 5 min exposure to each behavioral arena without any objects present. Twenty-four hours later, during the familiarization phase, a pair of identical novel objects were placed in each behavioral arena, and mice were allowed 5 min within each arena. Novel objects were always different between arenas, but identical within the same arena during the familiarization phase. At the end of the 5 min familiarization period in one arena, moveable doors were opened allowing the mouse to transition unaided to the next behavioral arena. After a familiarization period in each arena, mice were returned to their home cage to rest for 80 min. Following this, mice were reintroduced to the four behavioral arenas (in series) for the testing phase, which was identical to the familiarization phase except one object from each pair was swapped with a new object, now novel to the animal, and different from the objects in the remaining arenas. The behavior of the mice was monitored by an overhead camera, and exploration was analyzed offline (see Methods).

**Figure 2:**
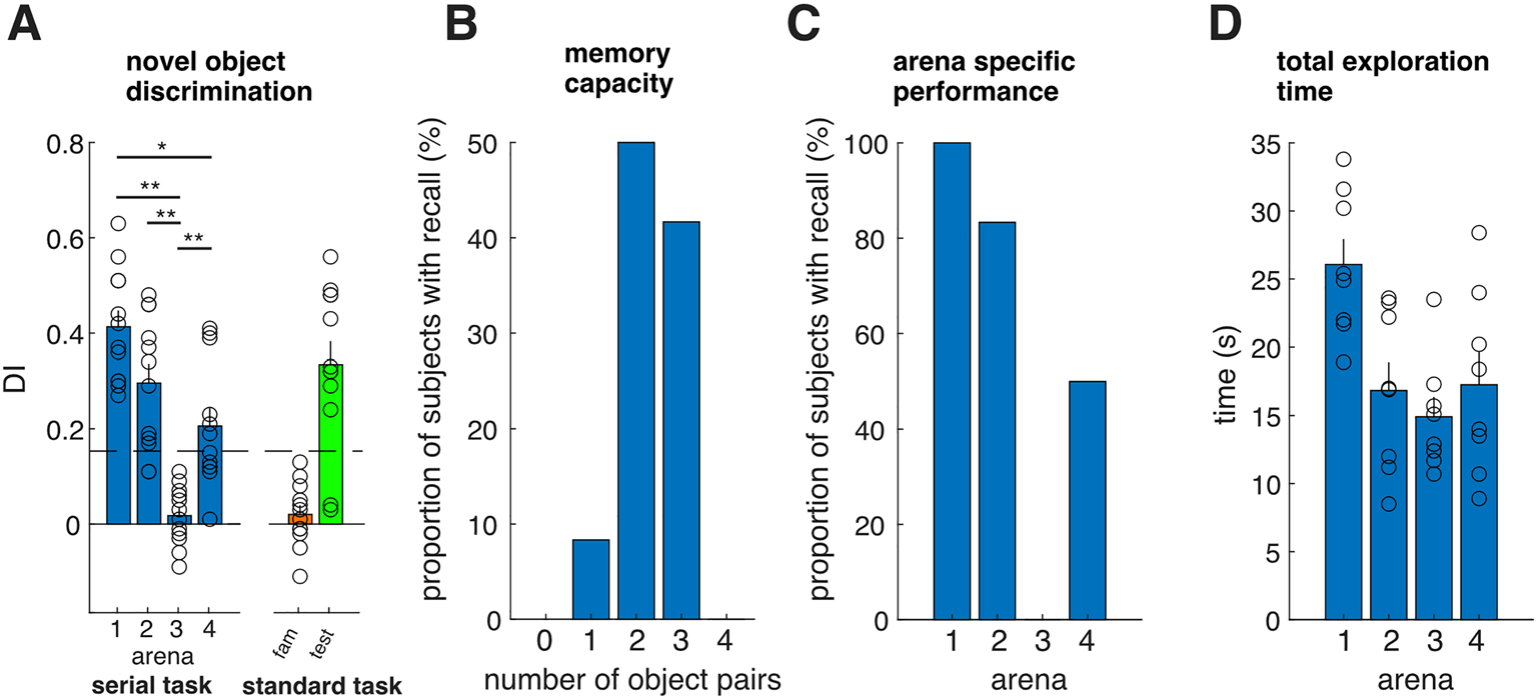
The memory capacity of mice. (**A**) Discrimination index in each arena (blue), compared to a single arena version of the task with 2 novel objects during the familiarization period (orange) or during the test period with one familiar and one novel object (green). (**B**) The distribution of the total number of object pairs remembered across subjects. (**C**) The proportion of subjects with a significant Discrimination index in each arena. (**D**) Total exploration time in each subject.

To measure memory retention in our serial recognition task, we computed the Discrimination Index (DI) [see Methods], which ranged from − 1 (only exploration of the familiar object) to 1 (only exploration of the novel object). We observed that the DIs in arena 1 and 3 tended to be the highest and lowest respectively (mean DI [arenas 1–4] = [0.41, 0.3, 0.012, 0.21], Fig. 2A), with a statistically significant difference in the DI between arenas 1 and 4 (*P* < 0.0205, Signed rank, Bonferroni corrected) and between arena 3 and each of the other three arenas (all *P* < 0.01, Signed rank test, Bonferroni corrected).

We next compared these results to a standard novel object discrimination task (single arena), during both the familiarization period (both objects were novel) and the testing phase (one object was familiar and one object was novel). Compared to the standard version of the novel object recognition task, involving a single arena and single pair of objects (Mean DI = 0.33), we did not observe any statistically significant differences in the DI for arenas 1, 2 and 4 (*P* > 0.05, Rank sum test, Bonferroni corrected). In contrast to this, arena 3 had a significantly lower DI compared to a single arena test (*P* < 0.0017, Rank sum test, Bonferroni corrected).

We next determined how many objects each subject could remember, with the threshold for successful recall being a discrimination index (DI) greater than a criterion of 0.153, equal to two standard-deviations above the mean of the DI distribution between two identical objects obtained during the single arena familiarization period (Fig. 2A, see Methods). We observed, qualitatively, that the majority of mice could remember 2–3 pairs of objects (Fig. 2B), with all mice successfully recalling the objects from arena 1, in contrast to none of the mice being able to remember the objects from arena 3 (Fig. 2C). No statistically significant difference in the total exploration time was observed between arenas 2, 3 and 4, with the total exploration time slightly greater for objects in arena 1 compared to the remaining arenas, albeit only with a statistically significant difference when compared with arena 2 and 3 (Signed rank test, Bonferroni corrected, *P* = 0.047 [vs. arena 2], *P* = 0.047 [vs. arena 3], Fig. 2D).

It is important to note that even when the memory capacity of the mouse was exceeded, the newly added items did not disrupt previously encoded memories (objects from arenas 1 and 2). While the serial position effect would predict the best memory recall for arenas 1 and 4, this was only partially true in our data; we observed a strong primacy effect (best performance in arena 1), but a weaker recency effect (50% of mice performed above chance in arena 4). Additionally, memory recall in arena 2 was not significantly different from arena 1 or 4 (*P* > 0.05, Sign ranked test, Bonferroni corrected).

**Figure 3:**
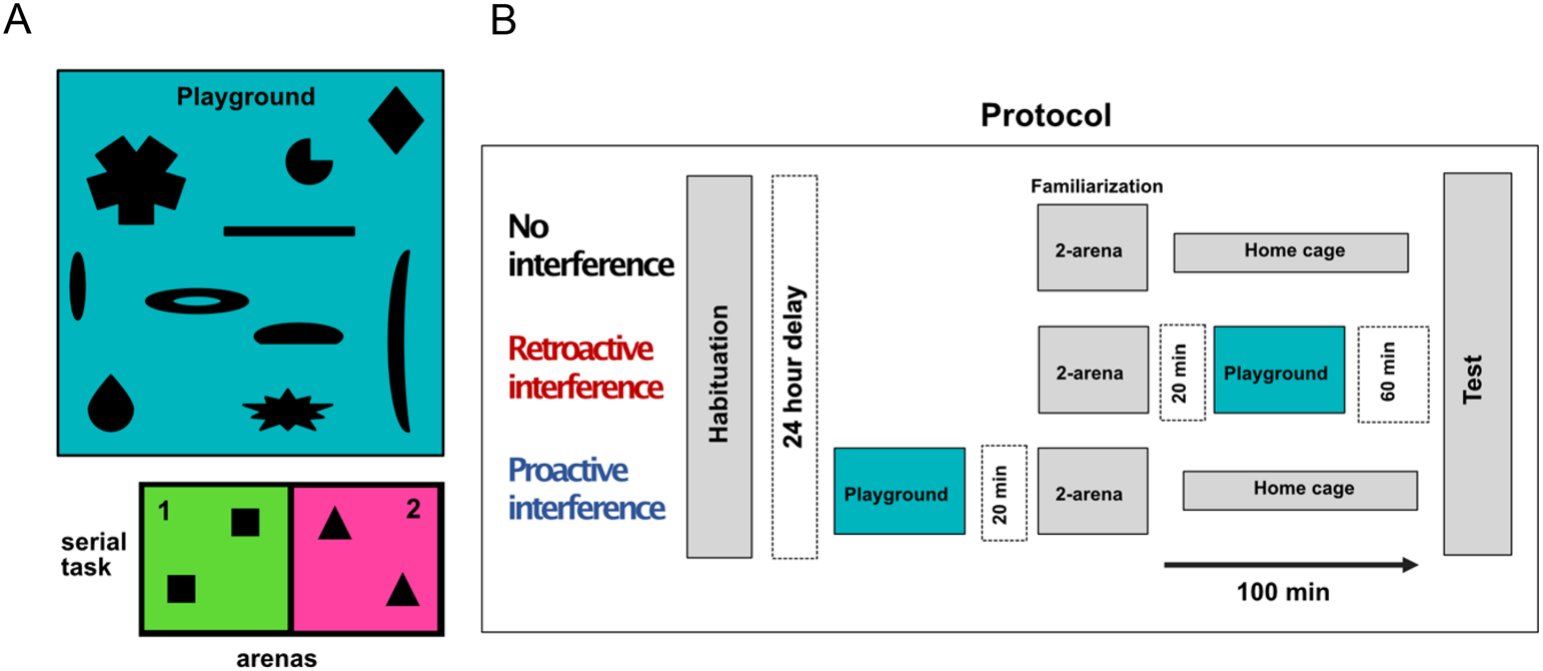
The protocol schematic and the performance of the NOR task with interference. (**A**) Schematic of the playground and two arenas in the salience NOR task. (**B**) Protocol for the variable salience NOR task in three groups containing the habituation phase, familiarization phase, testing phase and the playground. From the top to the bottom: no interference group, retroactive interference group, and proactive interference group, respectively.

We next investigated the effect of an interference task on memory recall, by including an additional arena (referred to here as “the playground”), which compared to the standard arena used was larger in size (> 11 times the area), had a longer exploration time (> 3 times the duration), and contained a greater number of novel objects (30 objects + 1 tube + 1 running wheel). The playground arena was explored either before or after the familiarization phase-referred to here as P_interference_ (proactive interference) and R_interference_ (retroactive interference), respectively [see Methods]. For comparison, we also compared these two groups with a standard serial novel objective discrimination task, which we refer to as the control group (no interference) (Fig. 3B). Given that most mice could remember 2 pairs of objects [see above], we only used two standard arenas for this version of the serial NOR task (Fig. 3A).

**Figure 4:**
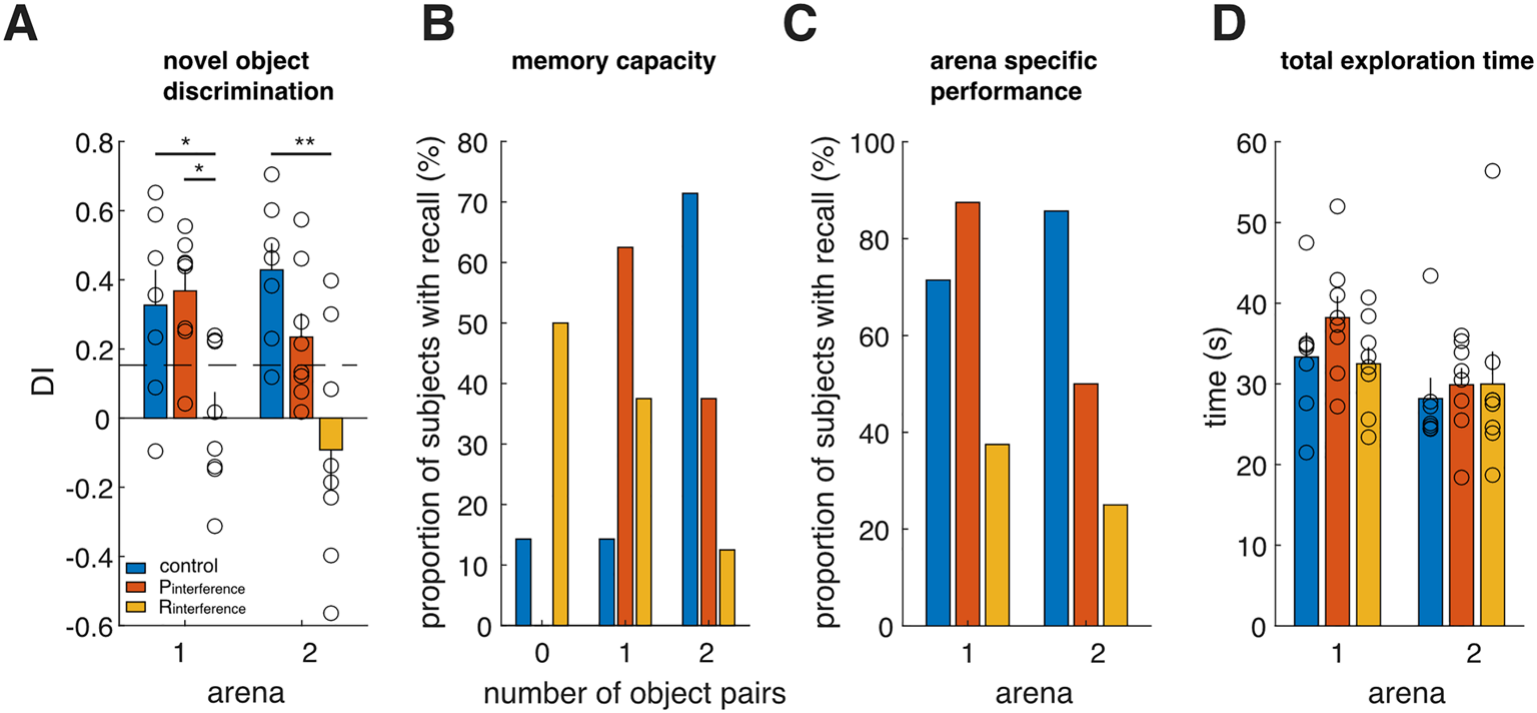
Effect of salience on the serial novel object discrimination task. (**A**) Discrimination index of no interference group (blue), proactive interference group (red) and retroactive interference group (yellow) in each arena. (**B**) The distribution of the total number of object pairs remembered across subjects. (**C**) The proportion of subjects with significant discrimination index in each arena. (**D**) Total exploration time in each subject for three groups in each arena.

We observed that in both arenas, the mean discrimination index of the R_interference_ mice was significantly impaired compared with the control group, while no statistically significant difference was observed between the P_interference_ and control group (Mean DI [arena 1,2]: (R_interference_ [0.002,-0.09], P_interference_ [0.37, 0.23], control [0.33, 0.43], Kruskal Wallis test with Bonferroni post hoc comparisons, R_interference_ versus control- [*P* = 0.0438; *P* = 0.0079], P_interference_ versus control- [*P* > 0.05, *P* > 0.05], Fig. 4A).

Similar to our analysis in the four-arena task, we next determined how many objects each subject could remember, with the threshold for successful recall being a discrimination index (DI) greater than a criterion of 0.153, equal to two standard-deviations above the mean of the DI distribution between two identical objects obtained during the single arena familiarization period. We observed, qualitatively, that the majority of mice remembered objects in both arenas for the control group, one arena from the proactive interference group, and neither arena from the retroactive interference group (Fig. 4B, see Methods). Interestingly, the task performance typically declined between the first and second arena for the proactive and retroactive-interference groups, while opposite trend was observed for the control group (memory recall was better for the second arena compared with the first arena) (Fig. 4C). No statistical difference in total exploration time was observed between arena 1 and 2 under all three interference conditions (Fig. 4D; arena 1: *p* = 0.2174; arena 2: *p* = 0.3817).

## Discussion

Here we describe a new behavioral paradigm that can test both the memory capacity and prioritization in rodents, using multiple novel object recognition tests performed serially in a multi-arena behavioural apparatus. Using this approach, we examined the effect of serial order and interference on memory retention.

A similar serial learning task has been previously reported in rats exploring a circular arena with multiple objects pairs distributed around the arena^21^. While such a paradigm has the advantage of not requiring any intervention from the experimenter, using a moveable wall panel (acting as a door) between arenas in our experiment allowed the researcher more precise control over the amount of time animals spent in each arena. Additionally, while our arenas areas were square to minimize any bias in where the mouse explored, the dimensions and shape could be easily modified, as well as the number of arenas visited, providing more flexibility in the experimental design.

Using a four-arena version of the task (Fig. 1), we observed that the memory capacity of female mice (for this specific task) is approximately 2–3 object pairs with a strong primacy effect (highest DI occurred in the first arena). Importantly, the introduction of additional arenas (each with a novel object pair) did not affect overall performance (measured as the discrimination index) compared to the standard version of the task performed using a single arena and object pair. This suggests that as memory demands increase beyond the brain’s capacity to consolidate, the brain’s strategy is to triage specific memories rather than try to remember everything at a lower fidelity. We do not know the true upper limit in memory in mice, as increasing the salience, exploration time, or delay period between arenas could potentially increase the number of objects remembered beyond our observations here. While observations are based on a serialized version of a novel object recognition task, a similar approach could be applied to other recognition tasks, including novel location and novel context, where engaging different brain regions for the task could lead to a different memory capacity^23,24^.

It is important to note that previous studies have demonstrated that memory performance can vary between male and female rodents. For example, Frick and Gresack^25^ report that male mice localize and recognize objects at a higher performance compared to female mice. Conversely it has also been reported that female rodents can perform better in spatial memory (object location)^26^. He we explicitly used female mice, which are generally under-used in behavioural studies, however as we did not test male mice, our results should not be generalized across sex. It is likely multiple factors beyond sex, including strain and age may also impact memory capacity and prioritization.

Introducing a new arena (“the playground”) with a longer exploration time and a higher number of novel objects interfered with memory retention in the 2-arena version of the serial novel object task. However, retroactive interference (exploration of the playground after familiarization in the 2-arena task) was more detrimental to memory retention than if the playground was explored before familiarization (proactive interference). This contrasts with our 4-arena task, where the first two arenas were generally remembered the best, with a similar memory performance to the single arena task, despite any possible retroactive interference from arenas 3 + 4. There are several possible reasons for this, including the potentially higher salience of the playground arena, the requirement of a higher memory load (more objects), as well as the longer delay period between the two arenas in the interference task. As we did not vary salience, exploration time and the delay period either independently or parametrically, the relative role and influence of these factors remain an open question.

Previous studies have suggested an opposite effect of novelty, in the form of ‘behavioural tagging’. If the encoding of a memory is sufficiently weak to only be remembered short-term but not long-term (i.e. successful recall after a 1 h but not a 24 h delay), this memory can be strengthened if paired with a second novel experience. For example, exploration of a novel open field close in time to a weakly-encoded experience modifies what would be only a short-term memory into a long-term memory. This memory strengthening effect of a novel experience can occur whether it occurs before or after the second experience forming the weak memory^27,28^, but requires the modified memory to be initially weak, and within a critical time window^29,30^. It is important to note that our experiments differed from a typical behavioural tagging paradigm in that (1) our memories were likely not weak, although we did not test them after a 24 h delay, and (2) our second novel experience was similar enough to create interference (both memories contained novel objects), and (3) mice were likely performing the task at or beyond their memory capacity. Nevertheless, why memory triage and behavioural tagging lead to seemingly opposing effects on memory as a consequence of novelty remains an open question.

What neural mechanisms are responsible for the prioritization and triage of memories^31^. One possibility is the emergence of post-training activity in specific brain regions, such as the basolateral complex of the amygdala (BLA). Post-training activity in the BLA has been previously shown to prioritize memory consolidation of specific object encounters^32–34^, including memory not typically considered to be related to emotional content, such as memory for novel objects in both rodents^35^ and human^36^. These studies have also indicated that the amygdala may not act alone, but in coordination with several brain regions including the hippocampus and perirhinal cortex. Another form of such activity that could prioritize memory consolidation is hippocampal replay, the spontaneous reactivation of memory traces during offline periods such as rest and sleep^37–42^. During sleep, more hippocampal replay is postulated to lead to memory strengthening while an insufficient amount of replay would result in memory triage (and eventual forgetting)^43^. Replay also occurs in the awake animal in the absence of locomotion (Foster and Wilson 2006)^44–46^, and this form of replay has recently been shown as a candidate mechanism for “tagging” salient memories for later sleep replay^47^. Because awake replay can occur remotely (while the animal is awake but not in the same context as the replayed behavioral episode), this may also help increase the likelihood of the memory later replaying during sleep.

## Methods

### Animals

C57BL/6J female mice (3 months of age) were used in all experiments. Mice were allocated to their home, four per cage, where were subject to a reverse light cycle (12 h/12 h dark/light cycle). All the mice were housed in their home cage for 10 days before the experiment with ad libitum access to food and water. No food or water restriction was performed before the behavioral tasks. All experimental procedures performed were first approved by a local ethical review committee at University College London. Procedures were carried out under license from the UK Home Office in accordance with the Animals (Scientific Procedures) Act 1986 under Project license-PPL P61EA6A72. All methods were also carried out in accordance with ARRIVE guidelines.

### Apparatus and materials

The four arena behavioral apparatus was 55 cm L × 55 cm W × 80 cm H, with an open-top field made of corrugated fluted board. The arena was constructed on a white table, with a surface made of particleboard. This provided a sufficient anti-slip surface during the behavioural task, while facilitating overhead image detection of the mouse vs. its background. This was used for all stages of the object recognition task. Barriers further subdivided the open field into four equal sub-areas (A, B, C, D) of approximately 27 cm × 27 cm (L × W) to create individual arenas. These barriers could be lifted individually, allowing the animal to walk to, and explore all relevant sub-area within a single session. The mean time spent in each arena was approximately 5 min [mean ± stderr for each arena across subjects: arena 1 (4.93 ± 0.06 min), arena 2 (4.96 ± 0.13 min), arena 3 (5.01 ± 0.08 min), arena 4 (5.04 ± 0.09 min)]. Using paper clips, large white shapes were hung down one wall of each sub-area to create four different contexts that could be easily discriminated by the mice. The room where the tasks were performed was approximately 3 m * 4 m (12 m^2^). All walls were plain white, and animals were not be able to see the room but only the arenas to minimise the potential effects. A 1W warm white bulb was located on the top of the task arena with a height of 1.5 m. It was in the center of the four arenas to make sure the light intensity on each arena floor was uniformly illuminated. The arena was only lightened by the bulb and no other light source in the room.

Two objects were placed diagonally (∼5 cm from the wall) in each context. Objects differed in height (2–10 cm), base diameter (2–6 cm), color, and shape. During the familiarization phase, two identical objects were presented in each arena, while during the testing phase, one of the familiar objects was replaced by a novel object. There were three copies of each object so that different copies of the same object could be presented during the familiarization and testing phases, to avoid the possibility of a residual odor on an object influencing the exploration time of the mouse. For all the experiments, objects and their relative positions were placed randomly and counterbalanced to avoid potential position effects in the arena. Pilot experiments were conducted to ensure mice could discriminate the different objects and did not show object preference.

For the playground arena, a 90 cm L × 90 cm W × 80 cm H open-top field made of corrugated fluted board was used to as “the playground” for the mice. Twenty individual or combined Lego blocks in five different colors, one wheel, one red tube and ten small colorful blocks were placed in the playground in a random arrangement. The layout of the playground remained the same for every mouse and was constant throughout the whole experiment. Every mouse was allowed to actively explore for no less than 15 min.

### Novel object recognition task

All NOR tasks were performed during the afternoon (1–6 pm). They were based on the following stages: (1) Habituation phase: 24 h before the familiarization, the mice were put into the apparatus for 5 min in each arena without the objects present (including the door-opening procedure). A similar habituation period was used for the playground arena. (2) Familiarization phase: mice were put in transfer cages with covers and transported from the colony arena to the experimental room 30 min prior to each familiarization phase to minimize stress related to their transportation. After this, animals were allowed to explore each arena (in the order of arenas 1 → 4) and its objects, for 5 min per arena. The interval period took place immediately after the familiarization session. Once the interval time elapsed the final testing phase of the NOR was conducted. (3) Testing phase: this phase was identical to the familiarization phase with the exception that one object in each context was replaced with a novel one. The mice were allowed to explore freely for 5 min in each arena. Any form of exploration beyond 5 min in each arena was not scored. The order of participation for group-housed mice was completely randomised. During the task, there were several separated empty cages in the experimental room, and mice were placed separately during tasks to avoid communicating with each other. They were not placed in the same cage nor back in their home cage before finishing all the tasks. The behavior of the mice was monitored by an overhead camera, and exploration was analyzed offline using the ANY-maze tracking system. To eliminate olfactory cues, after each phase, all the apparatus and objects were wiped and cleaned with water containing 50% ethanol, which remained the same for four arenas and the playground. Both experimenters (Q.Q. and C.M.) were female. The experimenter stayed at the far end of the room once the animal entered the arena to avoid any interaction. No other noise or procedure was performed when the video was recorded.

*Interference conditions.* 36 female mice were divided into three groups—two of them with a playground as memory interference before and after the familiarization phase, respectively. For the no interference group, mice were kept in their home cage during the entire interval time. For the groups with interference – mice were placed in the playground for 20 min, and in the resting cage for the remainder of the interval time. The total interval time for all three groups was 100 min (Fig. 3A).

*Automated tracking of object exploration.* Automated tracking of exploratory activity was conducted with ANYmaze software. For each video file, the 27.5 cm × 27.5 cm floor area of each arena and each object were outlined in ANYmaze as the main field. The head, body, and base of the tail of the mouse were automatically tracked by ANYmaze. Once a mouse satisfied the criteria for investigating an object, that period was accumulated to determine the total exploration time for a certain object. All files were coded to allow the experimenter to analyze the data blinded. We also analyzed videos manually (Hand-score) and checked the consistency between the software and the human observer. The results showed an extremely high similarity between ANYmaze and hand-scored (*r* = 0.97).

### Object investigating criteria

The criteria for active exploration for the mouse was its head was less than 20 mm from the object and oriented towards it^23,24^. Climbing over or leaning on an object was not considered to be an investigating behavior, unless that action was accompanied by a nose-directing behavior toward the object^48^. Our criteria for exploration also excluded time spent standing on top of objects^22^. Additionally, the 5 min time window in each arena started when the animal entered the arena. In most cases, once the door was opened, animals entered the next arena immediately.

### Data analysis

Memory performance on each recognition trial is expressed as the Discrimination Index (DI)

$$DI = \frac{{t_{novel} - \, t_{familiar} }}{{t_{novel} + \, t_{familiar} }}$$

where t_novel_ and t_familiar_ represent the total amount of time spent exploring novel and familiar object during the tasks respectively^22^. For the DI, the higher a ratio score is above zero (which indicates the mouse spent more time on the novel objects), the better the recognition memory performance. A significance level for the Discrimination index (0.153) was equal to two standard deviations above the mean of the DI distribution obtained during the familiarization period in the single arena version of the task. The proportion of subjects with recall (y-axis of Figs. 2B,C, 4B,C) is the proportion of subjects with a DI above the DI criterion of 0.153 (2 standard deviations above the mean DI of the familiarization session for a single arena). Custom scripts in MATLAB were used for data analysis.

## Data Availability

All data is available on Figshare-https://doi.org/10.6084/m9.figshare.22820450.v1. All code will be available via GITHUB (https://github.com/bendor-lab/serial_novel_object) upon publication.

## References

[1] Ebbinghaus, H. *Über das Gedchtnis. Untersuchungen zur experimentellen Psychologie*. Leipzig: Duncker & Humblot; the English edition is Ebbinghaus, H. (1913). *Memory. A Contribution to Experimental Psychology* (Teachers College, Columbia University (Reprinted Bristol: Thoemmes Press, 1999), New York, 1885).

[2] Greene AJ, Prepscius C, Levy WB (2000). Primacy versus recency in a quantitative model: activity is the critical distinction. Learn. Mem..

[3] Murdock Jr, Bennet B (1962). The serial position effect of free recall. J. Exp. Psychol..

[4] Daniel TA, Katz JS (2018). Primacy and recency effects for taste. J. Exp. Psychol. Learn. Mem. Cogn..

[5] Korsnes MS, Magnussen S, Reinvang I (1996). Serial position effects in visual short-term memory for words and abstract spatial patterns. Scand. J. Psychol..

[6] Miles C, Hodder K (2005). Serial position effects in recognition memory for odors: A reexamination. Mem. Cognit..

[7] Gaffan DA (1983). Comment on primacy effects in monkeys' memory for lists. Anim. Learn. Behav..

[8] Thompson RKR, Herman LM (1977). Memory for lists of sounds by the bottle-nosed dolphin: Convergence of memory processes with humans?. Science.

[9] Macphail EM (1980). Short-term visual recognition memory in pigeons. Q J. Exp. Psychol..

[10] Shimp CP (1976). Short-term memory in the pigeon: Relative recency. J. Exp. Anal. Behav..

[11] Bolhuis JJ, van Kampen HS (1988). Serial position curves in spatial memory of rats: Primacy and recency effects. Q. J. Exp. Psychol. Sect. B.

[12] Harper DN, Dalrymple-Alford JC, McLean AP (1992). Production of a serial position effect in rats using a 12-arm radial maze. J Neurosci Methods.

[13] Kesner RP, Novak JM (1982). Serial position curve in rats: Role of the dorsal hippocampus. Science.

[14] Reed P, Chih-Ta T, Aggleton JP, Rawlins JN (1991). Primacy, recency, and the von Restorff effect in rats’ nonspatial recognition memory. J. Exp. Psychol. Anim. Behav. Process..

[15] Zhan L, Guo D, Chen G, Yang J (2018). Effects of repetition learning on associative recognition over time: Role of the hippocampus and prefrontal cortex. Front. Hum. Neurosci..

[16] Crowley R, Bendor D, Javadi AH (2019). A review of neurobiological factors underlying the selective enhancement of memory at encoding, consolidation, and retrieval. Prog. Neurobiol..

[17] Foley NC, Jangraw DC, Peck C, Gottlieb J (2014). Novelty enhances visual salience independently of reward in the parietal lobe. J. Neurosci..

[18] Madan C (2017). Motivated cognition: Effects of reward, emotion, and other motivational factors across a variety of cognitive domains. Collab. Psychol..

[19] Skavronskaya L, Moyle B, Scott N (2020). The experience of novelty and the novelty of experience. Front. Psychol..

[20] Takeuchi T, Duszkiewicz AJ, Sonneborn A, Spooner PA, Yamasaki M, Watanabe M, Smith CC, Fernández G, Deisseroth K, Greene RW, Morris RGM (2016). Locus coeruleus and dopaminergic consolidation of everyday memory. Nature.

[21] Piterkin P, Cole E, Cossette MP, Gaskin S, Mumby DG (2008). A limited role for the hippocampus in the modulation of novel-object preference by contextual cues. Learn. Mem. (Cold Spring Harbor, N.Y.).

[22] Leger M, Quiedeville A, Bouet V, Haelewyn B, Boulouard M, Schumann-Bard P, Freret T (2013). Object recognition test in mice. Nat. Protoc..

[23] Dix SL, Aggleton JP (1999). Extending the spontaneous preference test of recognition: Evidence of object-location and object-context recognition. Behav. Brain Res..

[24] Langston RF, Wood ER (2010). Associative recognition and the hippocampus: Differential effects of hippocampal lesions on object-place, object-context and object-place-context memory. Hippocampus.

[25] Frick KM, Gresack JE (2003). Sex differences in the behavioral response to spatial and object novelty in adult C57BL/6 mice. Behav. Neurosci..

[26] Halpern D, Benbow CP, Geary DC, Gur RC, Hyde JS, Gernsbacher M (2007). The science of sex differences in science and mathematics. Psychol Sci Public Interest.

[27] Frey U, Morris RG (1997). Synaptic tagging and long-term potentiation. Nature.

[28] Rabinovich Orlandi I, Fullio CL, Schroeder MN, Giurfa M, Ballarini F, Moncada D (2020). Behavioral tagging underlies memory reconsolidation. Proc. Natl. Acad. Sci..

[29] Moncada D, Viola H (2007). Induction of long-term memory by exposure to novelty requires protein synthesis: Evidence for a behavioral tagging. J. Neurosci..

[30] Viola H, Ballarini F, Martínez MC, Moncada D (2014). The tagging and capture hypothesis from synapse to memory. Prog. Mol. Biol. Transl. Sci..

[31] Stickgold R, Walker MP (2013). Sleep-dependent memory triage: Evolving generalization through selective processing. Nat. Neurosci..

[32] Bass DI, Nizam ZG, Partain KN, Wang A, Manns JR (2014). Amygdala-mediated enhancement of memory for specific events depends on the hippocampus. Neurobiol. Learn. Mem..

[33] Bass DI, Manns JR (2015). Memory-enhancing amygdala stimulation elicits gamma synchrony in the hippocampus. Behav. Neurosci..

[34] Paré D (2003). Role of the basolateral amygdala in memory consolidation. Prog. Neurobiol..

[35] Roozendaal B, Castello NA, Vedana G, Barsegyan A, McGaugh JL (2008). Noradrenergic activation of the basolateral amygdala modulates consolidation of object recognition memory. Neurobiol. Learn. Mem..

[36] Inman CS, Manns JR, Bijanki KR, Bass DI, Hamann S, Drane DL, Fasano RE, Kovach CK, Gross RE, Willie JT (2018). Direct electrical stimulation of the amygdala enhances declarative memory in humans. Proc. Natl. Acad. Sci. USA.

[37] Girardeau G, Benchenane K, Wiener SI, Buzsáki G, Zugaro MB (2009). Selective suppression of hippocampal ripples impairs spatial memory. Nat. Neurosci..

[38] Lee AK, Wilson MA (2002). Memory of sequential experience in the hippocampus during slow wave sleep. Neuron.

[39] Silva D, Feng T, Foster DJ (2015). Trajectory events across hippocampal place cells require previous experience. Nat. Neurosci..

[40] Takigawa M, Huelin-Gorriz M, Tirole M, Bendor D (2022). Evaluating hippocampal replay without a ground truth. bioRxiv.

[41] Tirole M, Huelin Gorriz M, Takigawa M, Kukovska L, Bendor D (2022). Experience-driven rate modulation is reinstated during hippocampal replay. Elife.

[42] Wilson MA, McNaughton BL (1994). Reactivation of hippocampal ensemble memories during sleep. Science.

[43] Lewis PA, Bendor D (2019). How targeted memory reactivation promotes the selective strengthening of memories in sleep. Curr Biol CB.

[44] Carr MF, Jadhav SP, Frank LM (2011). Hippocampal replay in the awake state: A potential substrate for memory consolidation and retrieval. Nat. Neurosci..

[45] Diba K, Buzsáki G (2007). Forward and reverse hippocampal place-cell sequences during ripples. Nat. Neurosci..

[46] Foster DJ, Wilson MA (2006). Reverse replay of behavioural sequences in hippocampal place cells during the awake state. Nature.

[47] Huelin Gorriz M, Takigawa M, Bendor D (2023). The role of experience in prioritizing hippocampal replay. BioRxiv.

[48] Besheer J, Bevins RA (2000). The role of environmental familiarization in novel-object preference. Behav. Proc..

